# Low C6orf141 Expression is Significantly Associated with a Poor Prognosis in Patients with Oral Cancer

**DOI:** 10.1038/s41598-019-41194-1

**Published:** 2019-03-14

**Authors:** Cheng-Mei Yang, Hao-Sheng Chang, Hung-Chih Chen, Jyun-Jie You, Huei-Han Liou, Su-Chen Ting, Luo-Ping Ger, Sung-Chou Li, Kuo-Wang Tsai

**Affiliations:** 1 Department of Stomatology, Kaohsiung Veterans General Hospital, Kaohsiung, Taiwan; 2 Department of Dental Technology, Shu-Zen Junior College of Medicine and Management, Kaohsiung, Taiwan; 3 Department of Medical Education and Research, Kaohsiung Veterans General Hospital, Kaohsiung, Taiwan; 4 Planning Office of Kaohsiung Municipal United Hospital, Kaohsiung, Taiwan; 5 Institute of Biomedical Sciences, National Sun Yat-Sen University, Kaohsiung, Taiwan; 6 Genomics & Proteomics Core Laboratory, Department of Medical Research, Kaohsiung Chang Gung Memorial Hospital and Chang Gung University College of Medicine, Kaohsiung, Taiwan; 7 Department of Chemical Biology, National Pingtung University of Education, Pingtung, Taiwan

## Abstract

C6orf141 (Chromosome 6 open reading frame 141) is a novel gene, and its role in oral cancer progression remains unclear. C6orf141 expression in oral squamous cell carcinoma (OSCC) and adjacent normal tissues from 428 patients was examined through immunohistochemistry (IHC). Our results revealed that C6orf141 expression was significantly reduced in OSCC compared with adjacent normal tissues. Low C6orf141 expression was significantly associated with a poor American Joint Committee on Cancer pathological stage (*P* < 0.001), T classification (*P* = 0.002), and pN stage (*P* = 0.032). Kaplan–Meier curves revealed that low C6orf141 expression was significantly associated with shorter disease-specific survival (DSS) in patients with OSCC (log-rank *P* = 0.007). Multivariate analysis indicated that low C6orf141 expression was an independent prognostic biomarker for DSS (adjusted hazard ratio = 1.34; 95% confidence interval = 1.10–1.81; *P* = 0.05). Additionally, ectopic C6orf141 expression could significantly suppress oral cancer cell proliferation, colony formation, and migratory and invasive abilities. Xenograft tumor growth assay revealed that C6orf141 could significantly suppress oral tumor growth *in vivo*. Our results suggest that C6orf141 plays a novel tumor-suppressive role in oral cancer cell growth and motility. Furthermore, C6orf141 dysfunction could be a potential prognostic biomarker for OSCC and provide new therapeutic strategies in the future.

## Introduction

Oral cancer is the sixth most common malignancy worldwide^1,2^. It is the fourth cause of cancer death in male individuals and the top cancer in young male adults (25–44 years old) in Taiwan^3,4^. More than 90% of oral cancers are classified as oral squamous cell carcinoma (OSCC), typically observed on the tongue, buccal mucosa, and lips^5^. Habitual substance use, betel quid chewing, tobacco smoking, and alcohol drinking were reported to be associated with increased incidence and mortality rates of oral cancer^6^. Despite considerable progress in cancer treatment and management, the mortality rate associated with patients with OSCC remains unchanged. In Taiwan, the 5-year overall survival rate of patients with OSCC has remained at approximately 50% for several decades^4^. Metastasis and local recurrence are major problems due to therapy failure. However, most patients with OSCC were reported to be diagnosed at an advanced stage, leading to a low 5-year survival rate^3^. Therefore, developing a favorable biomarker for diagnosis or diagnosis using human genome information and molecular technologies may help to improve the survival rate of patients with OSCC.

Carcinogenesis is a complex multistep process in which genetic events within signal transduction pathways governing normal cellular physiology are quantitatively altered^7^. Gene dysfunctions are clinically attractive as candidate prognostic markers and therapeutic targets. Accumulating studies have identified several biomarkers for predicting OSCC progression, including moesin, Caspase 3, fibronectin 1, CD44, and SOX21-AS1^8–11^. In our previous study, we identified several deregulated genes in oral cancer through a next-generation sequencing approach^10^. Among these dysfunctional genes, chromosome 6 open reading frame 141 (C6orf141) located at chromosome 6p12.3 was identified as being involved in deregulating OSCC. However, the role of C6orf141 in human cancer is unknown.

According to the Human Protein Atlas (HPA) database, C6orf141 was expressed in some human cancers, including cancers of the brain, colon, duodenum, endometrium, esophagus, gall bladder, skin, stomach, and testis^12^. In addition, an analysis of The Cancer Genome Atlas (TCGA) database revealed that low expression levels of C6orf141 were associated with a poor survival rate in patients with breast cancer, endometrial cancer, and head and neck cancer^12^. However, the clinical effect of C6orf141 expression on OSCC remains largely unknown, and details of its role has not yet been fully elucidated. In this study, we examined the expression levels of C6orf141 in OSCC and adjacent normal tissues by using immunohistochemistry (IHC). Furthermore, we assessed the association between C6orf141 expression and clinical pathological features. We also assessed that effect of C6orf141 expression on cell growth and the invasion of OSCC cell lines.

## Results

### C6orf141 expression significantly decreased in OSCC

**Figure 1:**
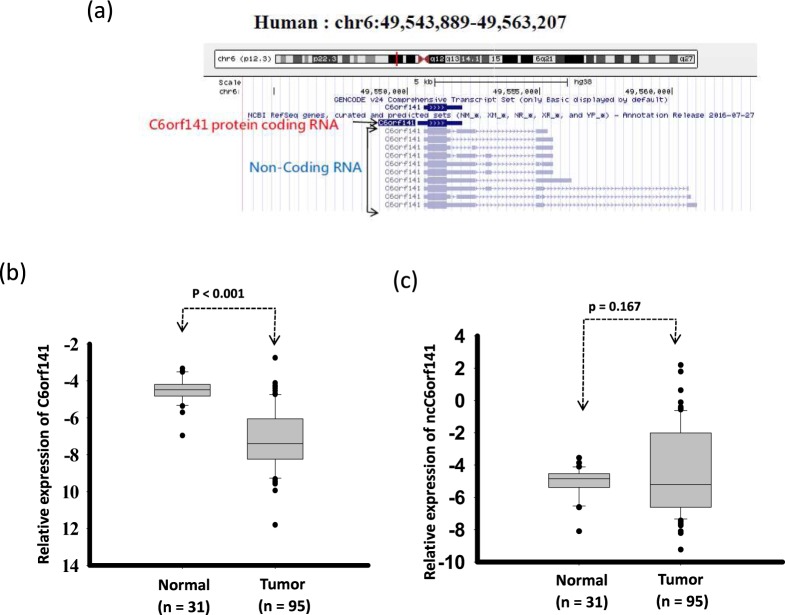
Expression levels of C6orf141 in oral cancer. (**a**) Schema of the locations of the C6orf141 and C6orf141 non-protein-coding genes determined using the UCSC Genome Browser of the human genome build GRCh37/hg19. (**b**) Expression levels of C6orf141 were examined in OSCC and adjacent normal tissues through real-time PCR. (**c**) Expression levels of noncoding transcripts of C6orf141 were examined using real-time PCR in OSCC and adjacent normal tissues.

C6orf141, located at 6p12.3, is a novel protein-coding gene that can generate a small protein with a molecular size of 26.8 kDa (Fig. 1a). According to the HPA database, C6orf141 is broadly expressed in human tissue, such as in the gastrointestinal tract, liver, and gallbladder^12^. The UCSC Genome Browser also reveals that C6orf141 can generate several noncoding RNA transcripts through alternative splicing (Fig. 1a). Therefore, we examined the expression levels of protein-coding and noncoding transcripts of C6orf141 in OSCC tissues from 95 patients by using real-time PCR. Our data revealed that the expression levels of the protein-coding transcripts of C6orf141 were significantly reduced, whereas those of noncoding transcripts had no significant difference in OSCC compared with adjacent normal tissue (Fig. 1b,c). We further examined the protein levels of C6orf141 in OSCC and adjacent normal tissues by using IHC. Of the 428 patients with OSCC examined in this study, 183 had buccal mucosa SCC (BMSCC) and 245 had tongue SCC (TSCC). In the cohort, 287 patients had tumor-adjacent normal tissues and 57 had normal uvula tissues. The clinical pathological features of patients with OSCC are summarized in Table 1.

**Table 1:** Clinicopathologic data of patients with OSCC.

Variable	OSCC for IHC	OSCC for Real-time PCR
	No. (%)	No. (%)
Sex		
Female	32 (7.5)	9 (9.5)
Male	396 (92.5)	86 (90.5)
Age, y		
≦40	71 (16.6)	6 (6.3)
41–50	136 (31.8)	18 (18.9)
51–60	123 (28.7)	37 (38.9)
>60	98 (22.9)	34 (35.8)
Subsite		
Buccal	183 (42.8)	36 (37.9)
Tongue	245 (57.2)	19 (20.0)
Other oral mucosal sites	0 (0.0)	40 (42.1)
Cell differentiation		
Well	73 (17.1)	14 (14.7)
Moderate	329 (76.9)	75 (78.9)
Poor	26 (6.1)	6 (6.3)
AJCC pathological stage		
I	135 (31.5)	19 (20.0)
II	143 (33.4)	28 (29.5)
III	65 (15.2)	6 (6.3)
IV	85 (19.9)	42 (44.2)
T classification		
T1	143 (33.4)	20 (21.1)
T2	188 (43.9)	36 (37.9)
T3	48 (11.2)	6 (6.3)
T4	49 (11.4)	33 (34.7)
N classification		
N0	331 (77.3)	70 (73.7)
N1 + N2	97 (22.7)	25 (26.3)

Abbreviations: SCC, squamous cell carcinoma; AJCC, American Joint Committee on Cancer.

**Figure 2:**
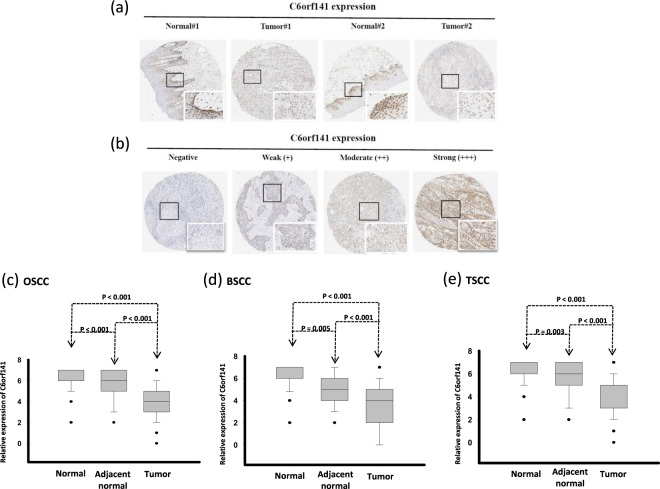
C6orf141 expression was significantly reduced in OSCC (**a**) Protein levels of C6orf141 in patients with OSCC were examined in OSCC tissues and corresponding adjacent normal tissues through IHC analysis, and representative images of 2 patients are presented. (**b**) IHC analysis of C6orf141 expression in the OSCC tissue microarray of 501 patients. Representative photomicrographs reveal negative (−), weak (+), moderate (++) and strong (+++) staining in OSCC tissues. (**c**–**e**) C6orf141 protein expression levels were measured and demonstrated a significant reduction in OSCC, BMSCC and TSCC tissues compared with corresponding adjacent normal tissues.

As depicted in Fig. 2a, the C6orf141 protein displayed major nuclear staining, and we observed a more drastic reduction in C6orf141 expression in OSCC tissues than in the corresponding adjacent normal tissues. We further analyzed tissue microarray data through scoring the intensity of C6orf141 staining. As presented in Fig. 2b, the intensity of C6orf141 was measured using a numerical scale (0, no expression; 1, weak expression; 2, moderate expression; and 3, strong expression). C6orf141 expression was lower in OSCC tissues (*P* < 0.001) than corresponding adjacent normal tissues (Fig. 2c and Table 2). C6orf141 downregulation was observed at different subsites of OSCC, revealing that C6orf141 expression was significantly lower in BMSCC and TSCC tissues than in corresponding adjacent normal tissues (Fig. 2d,e and Table 2; all *P* < 0.001). Furthermore, the expression levels of C6orf141 expression was also significantly attenuated in adjacent normal tissues compared with normal uvula tissues (Fig. 2c–e and Table 2).

**Table 2:** Immunohistochemical scores of c6orf141 in patients with OSCC, BMSCC, and TSCC.

Variables	Normal tissue		Tumor adjacent normal		Tumor		χ^2^	*p*-value*
	Mean ± SD	Median	Mean ± SD	Median	Mean ± SD	Median		
OSCC	(n = 57)		(n = 287)		(n = 428)			
	6.18 ± 1.15^g,h^	7.00	5.35 ± 1.45^g,i^	6.00	3.94 ± 1.81^h,i^	4.00	159.804	**<0.001**
BMSCC	(n = 27)		(n = 121)		(n = 183)			
	6.07 ± 1.17^a,b^	6.00	5.18 ± 1.54^a,c^	5.00	3.57 ± 1.89^b,c^	4.00	77.439	**<0.001**
TSCC	(n = 30)		(n = 166)		(n = 245)			
	6.27 ± 1.14^d,e^	7.00	5.47 ± 1.38^d,f^	6.00	4.22 ± 1.69^e,f^	5.00	84.914	**<0.001**
	Z = −0.872; p = 0.383		Z = −1.471; p = 0.141		χ^2^ = −3.517; p < 0.001			

Abbreviations: OSCC, oral squamous cell carcinoma; BMSCC, buccal mucosa squamous cell carcinoma; TSCC, tongue squamous cell carcinoma; SD, standard deviation.

*P values were estimated by Kruskal-Wallis one-way ANOVA test.

^a^P = 0.003; ^b^p < 0.001; ^c^p < 0.001; ^d^p = 0.001; ^e^p < 0.001; ^f^p < 0.001; ^g^p = 0.001; ^h^p < 0.001; ^i^p < 0.001.

### Effects of C6orf14 expression on clinicopathological outcomes of patients with OSCC

We further studied the association between C6orf141 expression and clinicopathological parameters including sex, age, cell differentiation, pathological stage, T classification, and N classification. As presented in Table 3, low C6orf141 expression was significantly associated with advanced pathological stage (I vs II + II + IV, *P* = 0.001), large tumor size (T1 vs T1 + 2 + 3, *P* = 0.002), and advance N stage (N0 vs N1 + N2, *P* = 0.032) in patients with OSCC, whereas a higher C6orf141 expression level was weakly correlated with older patients (*P* = 0.062). Additionally, C6orf141 expression in tumor tissues was significantly different among the BMSCC and TSCC (Table 3). C6orf141 expression was significantly lower in the buccal mucosal epithelium than in the tongue epithelium (Table 3, *P* < 0.001). Thus, a stratification analysis was performed according to the 2 different subsites. Our results reveal a significantly lower C6orf141 expression level along with advanced pathological stage (I vs II + III + IV, *P* < 0.001) and large tumor size (T1 vs T2 + 3 + 4, *P* < 0.005) in BMSCC, but not in TSCC (Table 4).

**Table 3:** The correlation between C6orf141 expression and clinical pathological features in patients with OSCC.

Variable	OSCC			
	No. (%)	Mean ± SD	Median	*p value*
Sex				
Female	32 (7.5)	4.41 ± 1.39	5.00	0.183^*^
Male	396 (92.5)	3.91 ± 1.83	4.00	
Age, y				
≦40	71 (16.6)	3.66 ± 1.94	4.00	**0.062** ^**†**^
41–50	136 (31.8)	3.79 ± 1.91	4.00	
51–60	123 (28.7)	3.98 ± 1.66	4.00	
>60	98 (22.9)	4.33 ± 1.69	5.00	
Subsite				
Buccal	183 (42.8)	3.57 ± 1.89	4.00	<0.001^*^
Tongue	245 (57.2)	4.22 ± 1.69	5.00	
Cell differentiation				
Well	73.1 (17.1)	3.99 ± 1.75	4.00	0.826^‡^
Moderate + Poor	355 (83.0)	3.94 ± 1.82	4.00	
AJCC pathological stage				
I	135 (31.5)	4.37 ± 1.70	5.00	**0.001** ^**‡**^
II + III + IV	293 (68.5)	3.75 ± 1.83	4.00	
T classification				
T1	143 (33.4)	4.33 ± 1.72	5.00	**0.002** ^**‡**^
T2 + T3 + T4	285 (66.5)	3.75 ± 1.82	4.00	
N classification				
N0	331 (77.3)	4.05 ± 1.78	4.00	**0.032** ^‡^
N1 + N2	97 (22.7)	3.60 ± 1.88	4.00	

Abbreviations: OSCC, oral squamous cell carcinoma; AJCC, American Joint Committee on Cancer.

*p values was estimated by Mann-Whitney U test.

^†^p values were estimated by Kruskal-Wallis one-way ANOVA test.

^‡^p values were estimated by student’s t-test.

**Table 4:** Correlation between C6orf141 expression and clinicopathological features in patients with BMSCC and TSCC.

Variable	BMSCC				TSCC			
	No. (%)	Mean ± SD	Median	*p value*	No. (%)	Mean ± SD	Median	*p value*
Sex								
Female	4 (2.2)	5.25 ± 0.96	5.50	0.072^*^	28 (11.4)	4.29 ± 1.41	5.00	0.839^*^
Male	179 (97.8)	3.53 ± 1.89	4.00		217 (88.6)	4.22 ± 1.73	5.00	
Age, y								
≦40	24 (13.1)	3.33 ± 2.18	4.00	0.087^†^	47 (19.2)	3.83 ± 1.81	4.00	0.138^†^
41–50	57 (31.1)	3.12 ± 1.91	3.00		79 (32.2)	4.27 ± 1.78	5.00	
51–60	56 (30.6)	3.77 ± 1.75	4.00		67 (27.3)	4.15 ± 1.58	4.00	
>60	46 (25.1)	4.00 ± 1.81	4.50		52 (21.2)	4.62 ± 1.54	5.00	
Cell differentiation								
Well	48 (26.2)	3.73 ± 1.85	4.00	0.495^*^	25 (10.2)	4.48 ± 1.45	5.00	0.427^*^
Moderate + Poor	135 (73.8)	3.51 ± 1.91	4.00		220 (89.8)	4.20 ± 1.72	5.00	
AJCC pathological stage								
I	64 (35.0)	4.25 ± 1.65^a^	5.00	**<0.001** ^**c**^	71 (29.0)	4.48 ± 1.75	5.00	0.133^*^
II + III + IV	119 (75.0)	3.20 ± 1.92	3.00		174 (71.0)	4.12 ± 1.66	4.00	
T classification								
T1	69 (37.7)	4.19 ± 1.70^b^	5.00	**<0.001** ^*^	74 (30.2)	4.46 ± 1.74	5.00	0.153^*^
T2 + T3 + T4	114 (38.3)	3.19 ± 1.91^b^	3.00		171 (69.8)	4.12 ± 1.67	4.00	
N classification								
N0	137 (74.9)	3.72 ± 1.81	4.00	0.057^*^	194 (79.2)	4.27 ± 1.73	5.00	0.381^*^
N1 + N2	46 (25.1)	3.11 ± 2.09	3.50		51 (20.8)	4.04 ± 1.56	4.00	

Abbreviations: BMSCC, buccal mucosa squamous cell carcinoma; TSCC, tongue squamous cell carcinoma; AJCC, American Joint Committee on Cancer.

*p values were estimated by student’s t-test.

^†^p values were estimated by one-way ANOVA test.

^c^p values were estimated by Mann-Whitney U test.

^a^p = 0.005; ^b^p = 0.007.

### Low C6orf141 was correlated with poor survival rates in patients with OSCC

**Figure 3:**
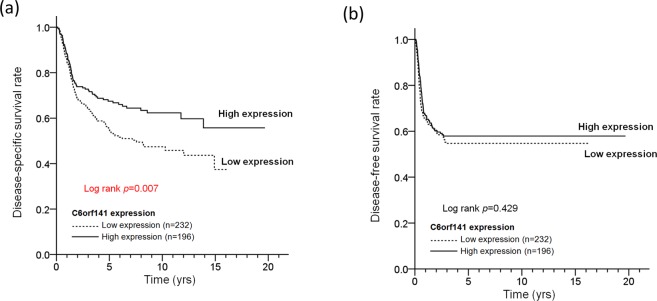
Low expression levels of C6orf141 were associated with a poor survival curve of patients with OSCC. (**a**,**b**) DSS and DFS were compared according to C6orf141 expression in OSCC by using the log-rank test.

**Figure 4:**
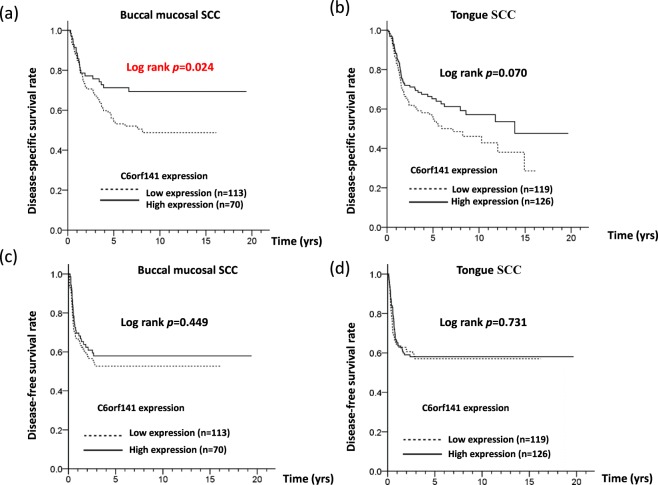
Prognostic significance of C6orf141 was analyzed in different types of OSCC. DSS and DFS were compared according to C6orf141 expression levels in BMSCC (**a**,**b**) and TSCC (**c**,**d**).

To determine whether C6orf14 is involved in the survival of patients with OSCC, a log-rank test was conducted, and Cox proportional hazards models were analyzed. First, we defined a cutoff value for C6orf121 levels, which was calculated using receiver operating characteristic (ROC) analysis. Based on this cutoff value, the patients were separated into two groups, which represented higher and lower C6orf141 expression in oral cancer. Our results showed that low C6orf14 expression was associated with an unfavorable DSS rate in patients with OSCC (log-rank test: *P* = 0.007, Fig. 3a), but the results revealed no significant difference in disease-free survival (DFS) (*P* = 0.429, Fig. 3b). A multivariate Cox’s regression model revealed a significant association of low C6orf141 expression with poor DSS (adjusted hazard ratio [AHR] = 1.34; 95% confidence interval [CI] = 1.10–1.81; *P* = 0.050), but the model indicated no significant with DFS (AHR = 1.10; 95% CI = 0.82–1.49; *P* = 0.513) (Table 5). A stratification analysis indicated that low C6orf14 expression was associated with poor DSS in patients with BMSCC (log-rank test: *P* = 0.024; Fig. 4a), but the results revealed no significant difference in DFS (Fig. 4b, *P* = 0.449). The analysis also showed a borderline significant association of low C6orf141 expression with poor DSS in patients with TSCC (Fig. 4c, *P* = 0.070) but no significant difference in DFS (Fig. 4d, *P* = 0.731). Further multivariate analysis revealed that low C6orf141 expression was associated with poor DSS in patients with BMSCC (AHR = 1.68; 95% CI = 1.02–2.79; *P* = 0.044), but the results indicated no correlation between DSS and TSCC (Table 5). These findings indicate that C6orf141 may play a crucial but different role in the clinicopathological outcomes of patients with OSCC, especially patients with BMSCC.

**Table 5:** Effect of C6orf141 expression on disease-specific survival and disease-free survival of patients with OSCC, BMSCC, and TSCC.

C6orf141 expression	NO. (%)	DSS		DFS	
		AHR^a^ (95% CI)	p-value	AHR^a^ (95% CI)	p-value
OSCC					
High	196 (45.8)	1	**0.05**	1.00	0.513
Low	232 (54.2)	1.34 (1.10–1.81)		1.10 (0.82–1.49)	
BMSCC					
High	70 (38.3)	1	**0.044**	1.00	0.514
Low	113(61.7)	1.68 (1.02–2.79)		1.17 (0.74–1.84)	
TSCC					
High	126 (51.4)	1	0.401	1.00	0.915
Low	119 (48.6)	1.18 (0.80–1.74)		1.02 (0.69–1.53)	

Abbreviation: OSCC, oral squamous cell carcinoma; BMSCC, buccal mucosa squamous cell carcinoma; TSCC, tongue squamous cell carcinoma; CI, confidence interval; AHR, adjusted hazard ratio.

^a^p-value were adjusted for T classification, N classification, cell differentiation.

### C6orf141 expression suppressed oral cancer cell growth and motility

**Figure 5:**
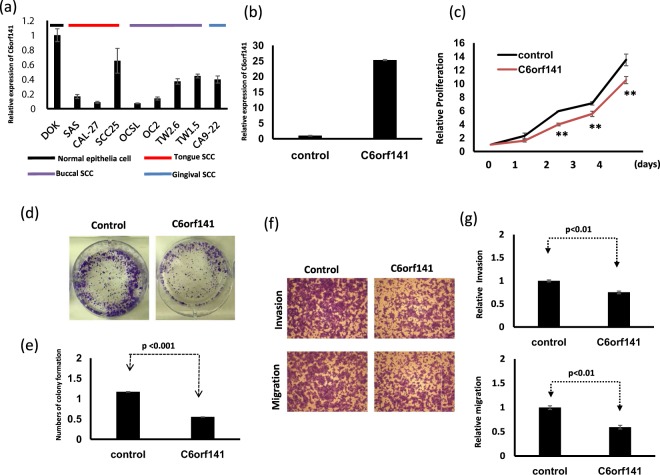
C6orf141 expression suppressed oral cancer cell growth and motility. (**a**) Expression levels of C6orf141 were examined in 8 oral cancer cell lines and a normal epithelial cell through real-time PCR. (**b**) Expression levels of C6orf141 were examined in SAS cells with pC6orf141 or vector transfection. (**c**) Cell proliferation was assessed in SAS with and without C6orf141 overexpression. (**d**) Colony formation assay was employed to examine SAS cells with and without C6orf141 overexpression. Cell photographs of a representative experiment are provided, and (**e**) data were quantified. (**f**,**g**) Migratory and invasive abilities were assessed using the Transwell assay in SAS cells with pC6orf141 or control transfection. Invading and migrating cells were stained with crystal violet solution, and the migration and invasion abilities were quantified through measuring 3 different fields under a phase-contrast microscope.

**Figure 6:**
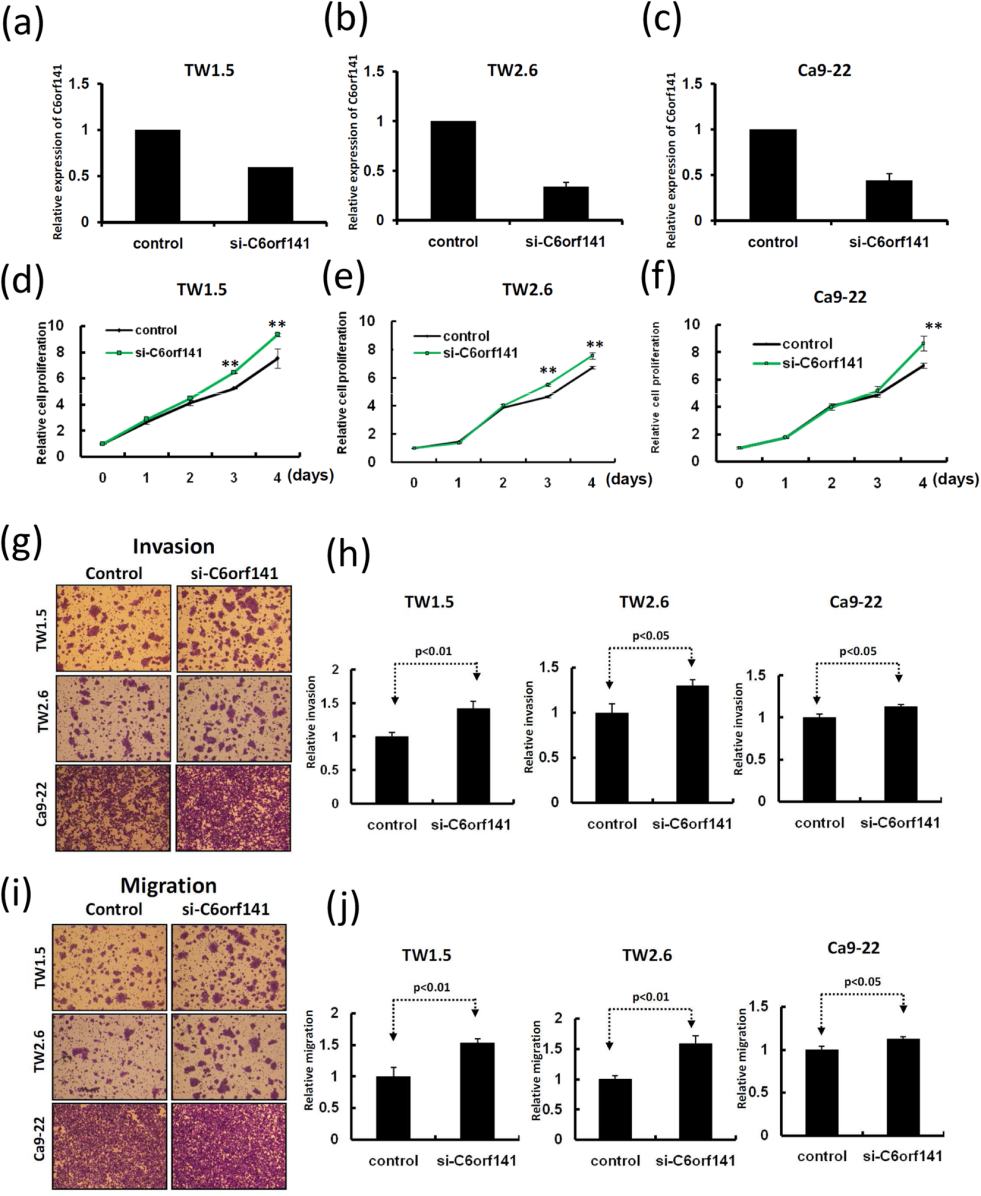
C6orf141 knockdown accelerated oral cancer cell growth and motility. (**a**–**c**) Expression levels of C6orf141 were examined in TW1.5, TW2.6, and Ca9-22 cells with siC6orf141 or scramble control transfection. (**d**–**f**) Cell proliferation was assessed in three cell lines with and without C6orf141 knockdown. (**g**,**i**) Invasive and migratory abilities were assessed using the Transwell assay in the three cell lines with siC6orf141 or control transfection. (**h**,**j**) Invading and migrating cells were stained with crystal violet solution, and the migration and invasion abilities were quantified through measuring three fields under a phase-contrast microscope.

We examined the expression levels of C6orf141 in eight oral cancer cell lines and the normal epithelial DOK cells through real-time PCR (Fig. 5a). Our data showed that C6orf141 expression was lower in all oral cancer cells than in DOK cells (Fig. 5a). Because C6orf141 expression was downregulated in oral cancer, gain of function might be a more effective approach to studying the biological function of C6orf141. In this study, we constructed a full-length C6orf141 expression vector, with a total length of 747 bp. We selected SAS, a cell line with a low expression level of C6orf141, for a series of functional assays using the gain-of-function approach. As depicted in Fig. 5b, the expression levels of C6orf141 were increased 25-fold in SAS with pC6orf141 transfection compared with the control group. Ectopic C6orf141 expression could significantly suppress oral cancer cell proliferation (Fig. 5c). A colony formation assay revealed that C6orf141 overexpression could also inhibit SAS cell colony formation (Fig. 5d,e). Furthermore, C6orf141 overexpression could significantly suppress SAS cell migration and invasion (Fig. 5f,g). We also selected TW1.5, TW2.6, and Ca9-22 cells, the cell lines with higher C6orf141 expression, for a series of functional assays by using the loss-of-function approach. Our data indicated that C6orf141 expression could significantly decrease in these cells with siC6orf141 transfection (Fig. 6a–c). Furthermore, C6orf141 knockdown could significantly accelerate oral cancer cell proliferation, migration, and invasion ability (Fig. 6d–j).

**Figure 7:**
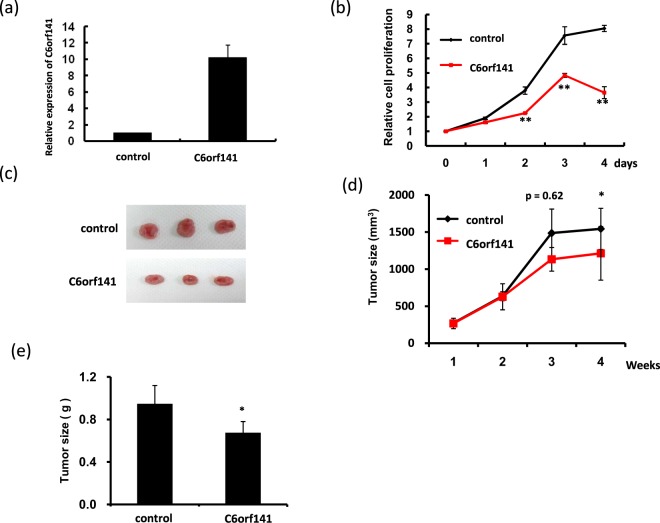
C6orf141 expression suppressed tumor growth in mouse models. (**a**) C6orf141 expression was examined in SAS cells with stable C6orf141 expression through real-time PCR. (**b**) Cell proliferation was assessed in SAS cells with or without stable C6orf141 expression. (**c**) The *in vivo* tumorigenic potential of C6orf141-overexpressing or control SAS cells were examined by subcutaneously injecting cancer cells into nude mice. (**d**,**e**) Volume and size of tumors were assessed.

**Figure 8:**
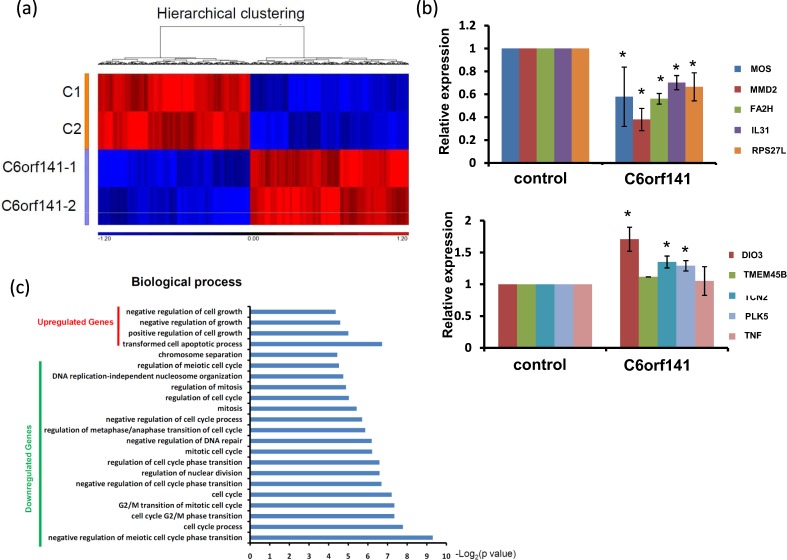
Transcriptome profiles of SAS cells with and without C6orf141 overexpression were analyzed through microarray. (**a**) Those genes in SAS cells with C6orf141 overexpression that were differentially expressed compared with control cells were identified. In total, 1130 significantly upregulated and 1176 significantly downregulated genes were identified. (**b**) The levels of 10 differential expression genes (5 downregulated genes in the upper panel and 5 upregulated genes in the lower panel) were examined in the SAS cells with and without C6orf141 expression through real-time PCR (*indicates p < 0.05). (**c**) Genes with differential expression were subjected to pathway enrichment analysis, and the cell cycle–related pathways were selected for presentation.

**Figure 9:**
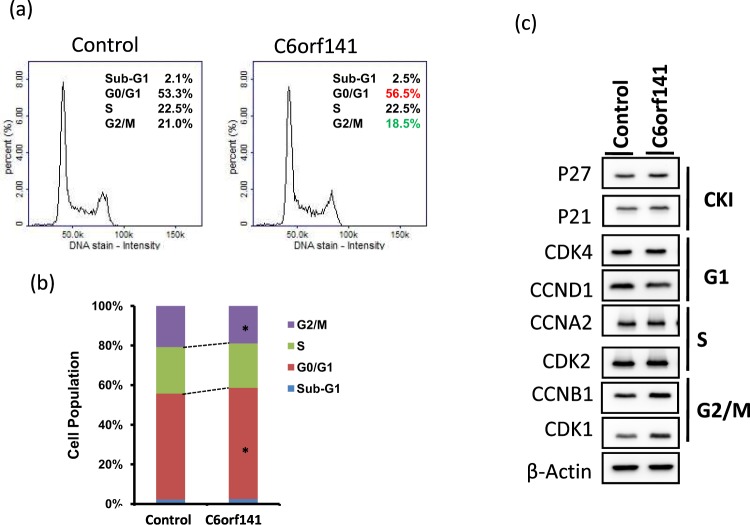
C6orf141 suppressed SAS cell growth by impairing cell cycle progression. (**a**) Distribution of SAS cells with and without C6orf141 overexpression in the three phases of the cell cycle was examined by image flow cytometry assay. (**b**) The graph shows quantification of each phase from three independent experiments. (**c**) The expression levels of the cell cycle-related genes in the SAS cells with and without C6orf141 expression were examined using Western blotting. *p < 0.05. The detail information of image was shown in supplementary data.

To assess the effects of C6orf141 expression on tumor growth *in vivo*, we generated SAS cells with stable C6orf141 expression. As shown in Fig. 7a,b, C6orf141 significantly increased, and proliferation was significantly suppressed in SAS cells with C6orf141 stably expressed. Xenograft tumor growth indicated that C6orf141 expression could significantly reduce tumor volume and size *in vivo* (Fig. 7c–e). We further performed RNA transcriptome of C6orf141 overexpression through microarray and identified 2306 genes in SAS cells with C6orf141 overexpression expressed differentially compared with control, including 1130 exhibiting upregulation and 1176 exhibiting downregulation (Fig. 8a). In order to verify the reliability of the microarray data, we further randomly selected 10 differentially expressed genes to confirm their expression levels in SAS cells with and without C6orf141 overexpression through real-time PCR, including 5 downregulated genes (MOS, MMD2, FA2H, IL31, and RPS27L) and 5 upregulated genes (DIO3, TMEM45B, TCN2, PLK5, and TNF). Except for TMEM45B and TNF, the expression levels of all genes were consistent with microarray results (Fig. 8b), implying that our microarray data was considerably reliable. A pathway enrichment analysis revealed that these differentially expressed genes were involved in several cell cycle-related signaling pathways (Fig. 8c). Furthermore, the image flow assay revealed that the proportion of the G0/G1 phase increased, whereas that of the G2/M phase decreased in the SAS cells with C6orf141 expression (Fig. 9a,b). We further examined cell cycle-related protein expression and found that the expression of cyclin D1 decreased, whereas that of p21, p27, CDK1 and CCNB1 increased in the SAS cells with C6orf141 overexpression (Fig. 9c). Our results implied that C6orf141 expression contributed to oral cancer cell growth by impairing cell cycle progression. In summary, our results indicate that C6orf141 may act a tumor suppressor in oral cancer. Low C6orf1411 expression may be a beneficial prognostic biomarker for OSCC.

## Discussion

OSCC is an aggressive malignancy and results in cancer deaths for male individuals in Taiwan^3,4^. Metastasis is a crucial problem due to therapy failure in patients with advanced-stage OSCC. Our study demonstrated that C6orf141, a novel cancer-related gene, has a tumor-suppressive role in OSCC cell growth and invasion. However, the mechanism of C6orf141 expression depletion in OSCC remains unknown. According to the Gene Expression Omnibus (GEO), the expression levels of C6orf141 could be silenced in breast cancer cell lines with ZNF217 (Zinc finger protein 217) overexpression^13^. ZNF217 was reported to play a crucial role in promoting breast cancer cell metastasis to bones^14^. Freier at al reported that 6.2% of patients with head and neck squamous cell carcinoma exhibited ZNF217 gene amplification^15^. These results suggest that C6orf141 downregulation might partially be a result of ZNF217 amplification in OSCC. In addition, through analyzing the GEO database, we observed that ectopic miR-34a and miR-335 expression could silence C6orf141 expression^16,17^. Previous studies have revealed that the expression levels of miR-335 were upregulated in the cancer-associated fibroblasts of patients with head and neck cancer. Furthermore, miR-335 expression could induce cancer cell motility through the suppression of phosphatase and tensin homolog signaling^18,19^. Perhaps, miR-335 suppressed cancer cell motility through inhibiting C6orf141 expression in OSCC. These potential mechanisms resulting in C6orf141 depletion in OSCC require further analysis in future works, including microRNA, and transcription factors.

Little information for C6orf141 on the roles in tumor-related function has been identified. There are some possible mechanisms to elucidate why C6orf141 underexpression occurs in OSCC. Cetuximab (Erbitux, Merck, Darmstadt, Germany) is a chimeric immunoglobulin (Ig) G1 monoclonal antibody directed against the epidermal growth factor receptor (EGFR). In combination with curative-intent radiotherapy, cetuximab has been reported to increase median survival in locally advanced head and neck carcinoma^20^. Together with some genes involved in tumor proliferation and inflammation, C6orf141 has been identified as significantly influenced by cetuximab^21^. Moreover, inhibition of OSCC development by C6orf141 may be achieved through the down-regulation of BRG1. C6orf141 was one of the downregulated genes in BRG1 knockdown cell lines. Previous studies have shown that BRG1 loss results in widespread changes in chromatin organization at regions including transcriptional start sites of cancer-associated genes and increased tumorigenic potential^22,23^.

Herein, we first report that C6orf141 protein was significantly reduced in OSCC tissues compared with adjacent normal tissues. An analysis of the relevant databases indicated RNA levels of C6orf141 to be inconsistent in different human cancers. The expression levels of C6orf141 were determined to be significantly reduced in thyroid cancer and testicular cancer, whereas those of C6orf141 were determined to be increased in colon cancer and lung cancer (data not shown). Furthermore, the effects of C6orf141 expression on the survival curve of patients with cancer were noted to be inconsistent. Based on TCGA database, high C6orf141 expression was noted to be significantly correlated with poor survival rates in patients with liver cancer, pancreatic cancer, kidney cancer, and gastric cancer. In contrast, high C6orf141 expression was observed to be associated with a more favorable survival curve than that of low C6orf141 expression in patients with head and neck cancer, breast cancer, ovarian cancer, and endometrial cancer. These results indicate that C6orf141 might have a distinct biological function in different cancer types. Our data also demonstrate that the expression levels of the protein-coding transcript of C6orf141 were clearly different from those of the noncoding transcripts of C6orf141 in patients with OSCC (Fig. 1b,c). The data of TCGA were collected through a next-generation sequencing approach; therefore, the inconsistent results might be due to inaccurate annotations. Furthermore, a previous study reported that the correlation coefficients between mRNA and protein concentrations are often remarkably low^24^. In multicellular organisms, the squared Pearson correlation coefficient (*R*^2^) ranges from 0.09 to 0.46. The low correlation between protein and mRNA levels results from translation efficiency, protein degradation, and mRNA stability. With these combined results, using mRNA to evaluate the clinical effects of C6orf141 in human cancer might result in some uncertainties. In the current study, we used protein levels of C6orf141 in OSCC to evaluate its clinical effects and observed that low C6orf141 was significantly associated with the prognosis of patients with OSCC. The specificity of aiti-C6orf141 antibody was confirmed using Western blotting (data not shown). Consistent with its clinical effects, ectopic C6orf141 expression could suppress oral cancer cell growth and invasion, implying that C6orf141 plays a tumor-suppressive role in OSCC progression. Previous studies revealed that the expression levels of tumor suppressor genes frequently were silenced with a DNA hypermethylated promoter^25^. In a human genome, a CpG-rich region is located upstream of C6orf141, and our results indicated that C6orf141 plays a tumor-suppressive role in oral cancer. Abe *et al*. also reported that promoter CpG islands of C6orf141 were methylated in neuroblastomas^26^. In addition, Jiang *et al*. reported that DNA methylation of C6orf141 has an interesting causal relationship with high-density lipoprotein changes^27^. Taken together, these results implied that the transcriptional activity of C6orf141 may be controlled through DNA methylation. We examined the relationships between the DNA methylation status of the CpG islands of C6orf141 and its expression levels. No methylation modification at the promoter regions of C6orf141 was observed in OSCC (data not shown). We also examined the expression levels of C6orf141 in oral cancer cells after 5-Aza-dC treatment. Results revealed that the expression levels of C6orf141 could not be increased in most oral cancer cell lines, except for SAS cells, after 5-Aza-dC treatment. Taken together, the results suggested that DNA methylation is not the major factor resulting in low C6orf141 expression in oral cancer progression.

In summary, we provide a novel insight that C6orf141 expression could suppress the growth and invasive abilities of OSCC cells. Furthermore, C6orf141 expression levels might serve as an unfavorable prognostic biomarker for patients with OSCC.

## Material and Methods

### Clinical samples

In this study, the collection of clinical samples was approved by the Institutional Review Board (IRB) of Kaohsiung Veterans General Hospital (KVGH) (IRB number: VGHKS14-CT6-18). A total of 95 OSCC tissues and 31 corresponding adjacent normal tissues were collected from patients with OSCC, who provided informed consent and underwent surgical operations at the Department of Dentistry and the Department of Otorhinolaryngology of KVGH. The methods were carried out in accordance with the approved guidelines and all patients provided informed consent. The clinical and pathological information of the patients is summarized in Table 1. All research was performed in accordance with relevant guidelines/regulations.

### RNA extraction

Total RNA was extracted using the TRIzol reagent (Invitrogen, Carlsbad, CA, USA), in accordance with the instruction manual. Briefly, tissue samples were first homogenized in 1 mL of TRIzol reagent, and then proteins and DNA were extracted using 0.2 mL of chloroform. Finally, total RNA was precipitated with 0.6 mL of isopropanol.

### Tissue microarray

Tissue microarrays containing 428 paraffin-embedded oral SCC cell samples were selected; these comprised 183 samples of buccal mucosal SCC and 245 samples of tongue SCC. The clinical and pathological information of the patients is summarized in Table 1. All patient data were obtained from the archives of the Department of Pathology of KVGH between 1990 and 2013. Normal uvula tissues were also collected from patients experiencing sleep apnea. These clinical samples were approved by the IRB (VGHKS14-CT6-18).

### Immunohistochemistry

In this study, a Novolink max polymer detection system (Leica Microsystems, Ltd., Milton Keynes, UK K) was used for IHC analysis. The slides were deparaffinized in xylene and rehydrated in grade alcohol. Antigen retrieval was performed by immersion in Tris-EDTA (10 mM, pH 9.0) for 10 minutes at 125 °C in a pressure boiler. Endogenous peroxidase activity was blocked through incubating the slides for 30 minutes with protein blocks (Novolink Polymer Detection Systems; Leica Microsystems, Ltd., Milton Keynes, UK). After blocking at room temperature (RT), primary antibodies were immediately applied and slides were incubated overnight at 4 °C in a wet chamber. The primary antibody used in this study was rabbit polyclonal anti-C6orf141 (1:200; Novus Biologicals; Littleton, Colorado, USA), in primary antibody diluent (Tris, Green; ScyTek Laboratories, Logan, UT, USA). After being washed in phosphate-buffered saline, the slides were incubated with a horseradish peroxidase-labeled secondary antibody for 10 minutes at RT, and the sections were counterstained with hematoxylin. For the antibody control studies, the serial sections were treated with phosphate-buffered saline, normal mouse lgG, normal rabbit IgG, and normal goat IgG, instead of the primary antibodies, and were confirmed to be unstained.

### Immunohistochemical analysis and scoring

At the beginning of the analysis, a senior pathologist accompanied a technician to evaluate the slides until all discrepancies were resolved. Then, the technician independently reviewed all slides. Finally, 5%20% core samples at each staining intensity were randomly selected for re-evaluation by the senior pathologist. During the evaluation, both the senior pathologist and the technician were unaware of the clinical outcomes of the patients. We graded the immunoreactivity through a semiquantitative approach. Marker scores for nuclear and cytoplasmic staining were calculated based on staining intensity (0, no signal; 1, mild; 2, moderate; and 3, strong) and on the proportion of positively stained tumor cells in 5 high-power fields (scored as 0, <5%; 1, 5–25%; 2, 26–50%; 3, 51–75%; and 4, >75%). The marker score was the sum of the staining intensity score and the percentage of positive tumor cell score. For survival analysis, C6orf141 expression levels were dichotomized as low expression and high expression with the cutoff set at the 50th percentile. The expression scores had an overall median score of 5.00 (range, 0–7), for C6orf141.

### Cell lines

Eight human oral cancer cell lines include three tongue SCC, (CAL27, SAS and SCC25) and four buccal SCC cells (OCSL, OC2, TW1.5, TW2.6), a gingival SCC cell (CA9-22), and a normal epithelia cells (DOK) were used in this study. The cells were maintained in Dulbecco modified Eagle medium (DMEM; Biological Industries USA, Cromwell, CT, USA), supplemented with 10% fetal bovine serum (FBS; HyClone; GE Healthcare Life Sciences, Logan, UT, USA) and penicillin–streptomycin (penicillin, 100 U/mL; streptomycin, 100 μg/mL; Sigma-Aldrich; Merck KGaA, Darmstadt, Germany) in a humidified atmosphere containing 5% CO_2_ at 37 °C.

### Reverse transcription polymerase chain reaction of RNAs

For reverse transcription polymerase chain reaction (PCR), 2 μg of total RNA was reverse transcribed using random primers and SuperScript III Reverse Transcriptase, according to the manufacturer instructions (Invitrogen; Carlsbad, CA, USA). Gene expression was detected using an SYBR Green I assay (Applied Biosystems, Foster City, CA), and C6orf141 expression was normalized to that of Glyceraldehyde 3-phosphate dehydrogenase (GADPH; △Ct = target C6orf141 Ct-S26 Ct). The individual primers were shown in Supplementary Table 1.

### C6orf141 expression construction

The full length of C6orf141 was synthesized and digested with the restriction enzyme EcoRI and SpeI. Then, the full length of C6orf141 was cloned into the pLVX-IRES-Neo vector (Clontech, Mountain View, CA, USA). Stable SAS cells with C6orf141 expression were generated by transfecting oral cancer SAS cells with pLVX-C6orf141 for 48 h followed by G418 (1200 μg/mL) selection for 14 days. Subsequently, overexpression efficiency was evaluated through real-time PCR.

### C6orf141 knockdown with siRNA

Small interfering RNA (siRNA) oligonucleotides targeting C6orf141 (si-sense: 5′-CUAAUGAGUAGCUCGAGAAdTdT-3′ and antisense: 5′-UUCUCGTGCUACUCAUUAGdTdT-3′) and a scrambled oligonucleotide as a negative control were designed and synthesized by SIGMA (Sigma–Aldrich, St. Louis, MO, USA). Oral cancer cells were transfected with a 10 mM (final concentration) siC6orf141 or scrambled control using Lipofectamine RNAiMAX (Invitrogen, Thermo Fisher Scientific, Carlsbad, CA, USA). After transfection for 24 h, knockdown efficiency was evaluated through real-time PCR.

### Colony formation and cell proliferation assay

For the clonogenic assay, a gradient number of SAS cells (2000, 4000, and 8000) were seeded in 6-well plates and transfected with a C6orf141 construct or a scramble control as aforementioned. The cells were incubated in a CO_2_ incubator at 37 °C for 2 weeks until colony formation. The cells were then fixed and stained with crystal violet solution (0.05% crystal violet, 1% formaldehyde, and 1% methanol) (Sigma-Aldrich, St. Louis, MO, USA) for 20 minutes at RT. Subsequently, the colony formation was determined using a microscope (×100 magnification; CKX41; Olympus Corporation, Tokyo, Japan). For the cell proliferation assay, 1.5 × 10^3^ SAS, TW1.5, TW2.6 and Ca9-22 cells transfected with C6orf141 expression vector, siC6orf141 or a scramble control, were seeded in a 96-well plate. Proliferation was determined at 0, 1, 2, 3, and 4 days using the CellTiter-Glo One Solution assay, according to the manufacturer’s instructions (Promega Corporation, Madison, WI, USA). All experiments were independently repeated 3 times.

### Cell migration and invasion assays

The oral cancer cells were analyzed using Transwell assays. Briefly, transfected cells at a density of 1.0 × 10^5^ were resuspended in DMEM (Biological Industries USA, Cromwell, CT, USA) with 2% FBS supplemented with penicillin–streptomycin (penicillin, 100 U/mL; streptomycin, 100 μg/mL; Sigma-Aldrich; Merck KGaA, Darmstadt, Germany). The cells were then added to the upper chamber of the Transwells (Falcon, Corning Incorporated, USA) without Matrigel (BD Biosciences, MA) for the migration assay or Matrigel coating for the invasion assay. Subsequently, DMEM (Biological Industries USA, Cromwell, CT, USA) supplemented with 10% FBS (HyClone; GE Healthcare Life Sciences, Logan, UT, USA) and penicillin–streptomycin (penicillin, 100 U/mL; streptomycin, 100 μg/mL; Sigma-Aldrich; Merck KGaA, Darmstadt, Germany) was added to the lower chambers for the invasion assay. The chambers were incubated in a CO_2_ incubator at 37 °C for 24 or 48 hours. The remaining cells in the upper chamber were then removed using cotton swabs, and the cells under the surface of the Transwell plates were fixed with 4% formaldehyde solution for 10 minutes at RT. The cells were stained with crystal violet solution (0.05% crystal violet, 1% formaldehyde, and 1% methanol) (Sigma-Aldrich, St. Louis, MO, USA) for 20 minutes at RT, and the oral cancer cells in the 3 fields of view were counted through phase-contrast microscopy. All experiments were repeated 3 times.

### Xenograft tumor growth assay

Animal experiments were approved by the Kaohsiung Veterans Hospital Laboratory Animal Center and Use Committee. Nude mice (4 weeks old) were used in this study; 2 × 10^6^ SAS cells with C6orf141 stable expression or control cells were suspended in PBS and implanted on the backs of nude mice. Animal were observed, and tumor volume was evaluated as *V* (in mm^3^) = largest length × 0.52 × (shortest length)^2^ each week for 4 weeks. At 4 weeks after implantation, the animals were sacrificed, and tumor size and weight were assessed. All research was performed in accordance with relevant guidelines/regulations

### Microarray analysis and pathway enrichment analysis

SAS cells with C6orf141 overexpression and control cells were used as total RNA sources. The RNA samples were first examined with TapeStation 4200 to ensure that their RNA integrity number (RIN) values reached up to 7, followed by preparation with WT PLUS reagent (Invitrogen, Thermo Fisher Scientific, Carlsbad, CA, USA). Then, the prepared samples were hybridized on human Clariom D microarray chips (Invitrogen, Thermo Fisher Scientific) and scanned using a GeneChip Scanner 3000 7 G (Invitrogen, Thermo Fisher Scientific, Carlsbad). The microarray raw data passing quality control were analyzed using Partek (Qiagen Sciences®, Germantown, MD, USA) to identify differentially expressed genes (p < 0.05). As a result, we identified 2,306 significant genes, as illustrated in the heat map figure, among which 1,130 and 1,176 genes were upregulated and downregulated, respectively. All microarray data were submitted to the NCBI GEO and are freely available with the accession number GSE123456. We determined whether the functions of the C6orf141-regulated genes were involved by investigating the pathways. The differentially expressed genes were selected from the microarray data; subsequently, candidate genes were mapped onto Kyoto Encyclopedia of Genes and Genomes (KEGG) pathways.

### Cell cycle analysis

A total of 1 × 10^6^ cells were collected and mixed with 70% ethanol in a fixative and incubated at −20 °C overnight. The cells were then stained with 4′,6-diamidino-2-phenylindole (ChemoMetec, Gydevang, Lillerød, Denmark) and detected by the NucleoCounter NC-3000 and analyzed using NucleoView NC-3000 software (ChemoMetec).

### Western blotting

The SAS cells with and without C6orf141 expression were harvested 24 h after transient transfection. Total protein was collected and subjected to Western blot analysis. Total protein were separated by 6%-10% sodium dodecyl sulfate–polyacrylamide gel electrophoresis and transferred onto nitrocellulose filter membranes (Millipore, Billerica, USA). Finally, the proteins were visualized using WesternBright^TM^ ECL (Advansta Inc., Menlo Park, CA, USA) and detected using the BioSpectrum^TW^ 500 Imaging System (UVP, USA). The detailed information has been described in our previous study^28^. In this study, the following primary antibodies were used: CCNA2 (1:1000; 18202-1-AP, Proteintech Group, Inc., Rosemont, IL, USA), CCNB1(1:1000; 55004-1-AP, Proteintech Group, Inc.,), CCND1 (1:1000; RM-9104-S, Thermo Fisher Scientific Inc., Waltham, MA, USA), CDK4 (1:1000; MS-299-P, Thermo Fisher Scientific Inc., Waltham), CDKN1B (1:1000; #3686, Cell Signaling Technology, Inc., Beverly, MA, USA), CDKN1A (1:1000; #2947, Cell Signaling Technology, Inc.,), and ACTB (1:2000, MAB1501, EMD Millipore, Billerica, MA, USA).

### Statistical analysis

The chi-squared test, Fisher exact test, Student *t* test, analysis of variance (ANOVA), Mann–Whitney U test, or Kruskal–Wallis one-way ANOVA was used to evaluate the correlation of C6orf141 expression with different oral tissues or clinicopathological parameters. Clinicopathological outcome is usually defined as the time from initial diagnosis or surgery to a specific event of interest. Disease-specific survival (DSS) was measured from the time of the initial resection of the primary tumor to the date of the cancer-specific death or last follow-up. Cumulative survival curves were estimated using the Kaplan–Meier method, and comparisons between the survival curves were conducted using the log-rank test. A Cox proportional hazards model was used to determine independent predictors of survival using factors that were deemed significant in the univariate analysis as covariates. A *P* value of <0.05 was considered statistically significant.

## Supplementary information

Supplementay table and information of figure 9c
