# Supplementary material for: Low C6orf141 Expression is Significantly Associated with a Poor Prognosis in Patients with Oral Cancer
Source: Sci Rep. 2019 Mar 14;9:4520. doi: 10.1038/s41598-019-41194-1 (PMC6418188; doi:10.1038/s41598-019-41194-1)
Supplement: Supplementary file 1 — Supplementay table and information of figure 9c [file 41598_2019_41194_MOESM1_ESM.pdf]

Low C6orf141 Expression Is Significantly Associated With a Poor Prognosis in Patients With Oral Cancer

Cheng-Mei Yang[1,2], Hao-Sheng Chang[1,2], Hung-Chih Chen[1,2], Jyun-Jie You[3], Huei-Han Liou[3], Su-Chen Ting[4], Luo-Ping Ger[3,5], Sung-Chou Li[6$], Kuo-Wang Tsai[3,5,7$]

[1] Department of Stomatology, Kaohsiung Veterans General Hospital, Kaohsiung, Taiwan

[2] Department of Dental Technology, Shu-Zen Junior College of Medicine and Management, Kaohsiung, Taiwan

[3] Department of Medical Education and Research, Kaohsiung Veterans General Hospital, Kaohsiung, Taiwan

[4] Planning Office of Kaohsiung Municipal United Hospital, Kaohsiung, Taiwan

[5] Institute of Biomedical Sciences, National Sun Yat-Sen University, Kaohsiung, Taiwan

[6]Genomics & Proteomics Core Laboratory, Department of Medical Research, Kaohsiung Chang Gung Memorial Hospital and Chang Gung University College of Medicine, Kaohsiung, Taiwan

[7]Department of Chemical Biology, National Pingtung University of Education, Pingtung, Taiwan

Correspondence: Kuo-Wang Tsai, Department of Medical Education and Research, Kaohsiung Veterans General Hospital, Kaohsiung 813, Taiwan. Tel: 011-886-7-342-2121 ext. 1510, Fax: 011-886-7-346-8056, E-mail: kwtsai6733@gmail.com and Sung-Chou Li, Genomics & Proteomics Core Laboratory, Department of Medical Research, Kaohsiung Chang Gung Memorial Hospital and Chang Gung University College of Medicine, Kaohsiung, Taiwan. Tel:011-886-7-7317123 ext.8156, E-mail: raymond.pinus@gmail.com

Running title: C6orf141 Is a Novel Tumor-Suppressor Gene in Oral Cancer

Supplementary Table 1. The primers information

name	sequence
**C6orf141-F**	5'-AGGAGCCCAACTACCCTTCT-3'
**C6orf141-R**	5'-TCCTCAGTCCTCGTGGTCAT-3'
**C6orf141-ncRNA-F**	5'-GGTGGATGAACCGCATCAAAG-3'
**C6orf141-ncRNA-R**	5'-GATCCTGGGGGTTATGCGAT-3'
**GAPDH-F**	5'-TGCACCACCAACTGCTTAGC-3'
**DAPDH-R**	5'-GGCATGGACTGTGGTCATGAG-3'
**DIO3-F**	5'-TCACTAAGTACCAGCGCGAC-3'
**DIO3-R**	5'-CTCGAAGTAGGCGCCATAGG-3'
**TMEM45B-F**	5'-TGAGGAATGAGCCGAGATGC-3'
**TMEM45B-R**	5'-AAGATCAGACCACAGCAGGC-3'
**TCN2-F**	5'-AACAGCATGTCTCAAGGCGA-3'
**TCN2-R**	5'-GTACGGCGGCAAGAGACTAA-3'
**TBX15-F**	5'-CAAGCCCGGAAAAACTGAGC-3'
**TBX15-R**	5'-GGGGCCTTGATTGCCAAATG-3'
**PLK5-F**	5'-CGCCTTCCTGCGAGACC-3'
**PLK5-R**	5'-TGGTGGACATGTCTGTCAGC-3'
**TNF-F**	5'-TGAATGTATTTATTTGGGAGACCG-3'
**TNF-R**	5'-ACAGACACAACTCCCCTGG-3'
**MMD2-F**	5'-CAAGAGGTACCAGCCCACAG-3'
**MMD2-R**	5'-CACAGTGGACACCACGAAGA-3'
**FA2H-F**	5'-AGAAGAGACAGCGTGATGCC-3'
**FA2H-R**	5'-TGGTGGAGAAGCCTCCTCAG-3'
**IL31-F**	5'-GAAGCTGGCCTTGCTCTCTC-3'
**IL31-R**	5'-CTGTGGATGTTGTTTGGCGG-3'
**MOS-F**	5'-ATTGACTGGGAGCAGGTGTG-3'
**MOS-R**	5'-CTAGGCTATTGGACCCTGCG-3'
**RPS27L-F**	5'-TAACTGCTAGCTGGGGTGTG-3'
**RPS27L-R**	5'-AGGCACCAGAACCACTCAAC-3'

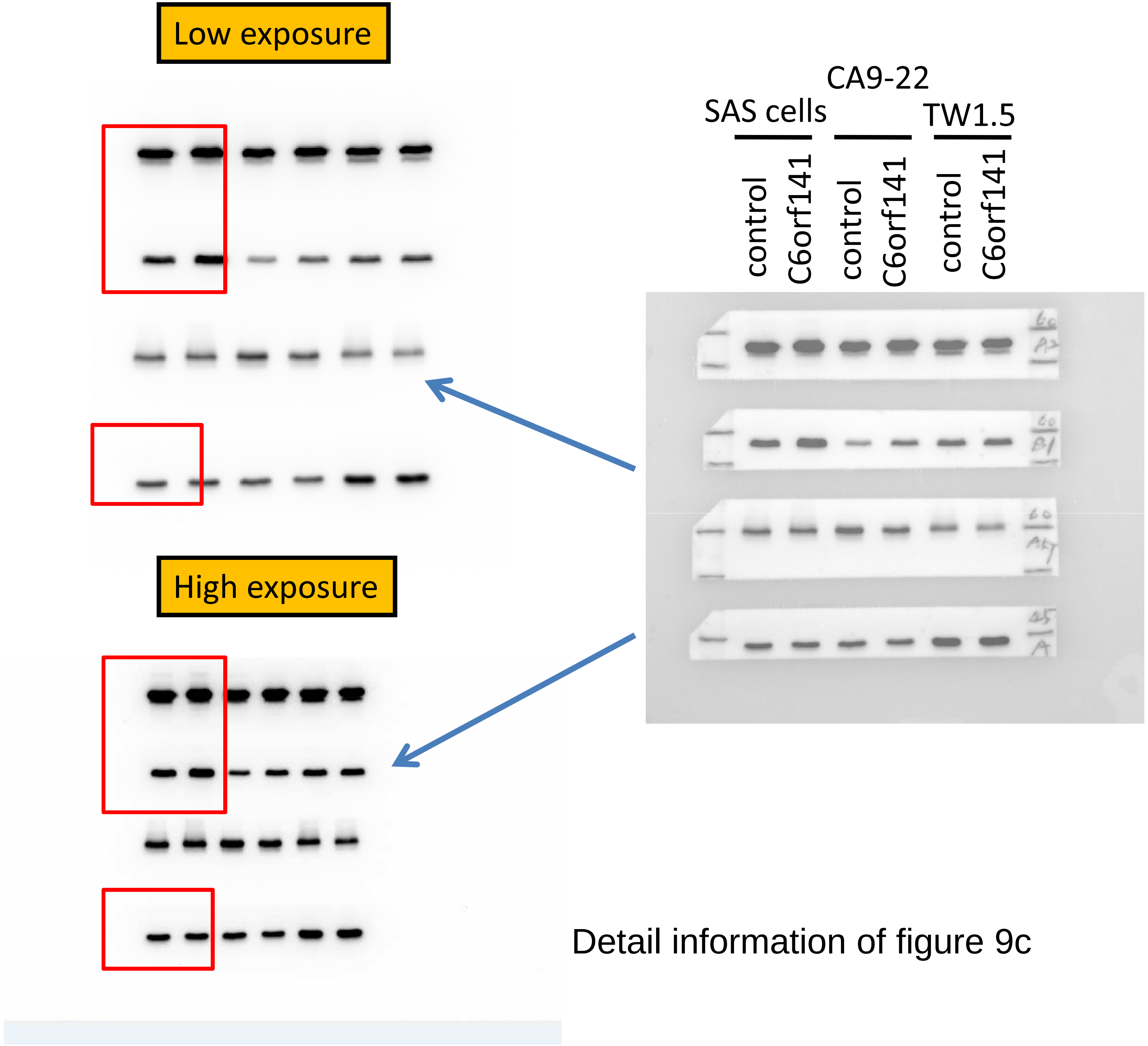

Detail information of figure 9c

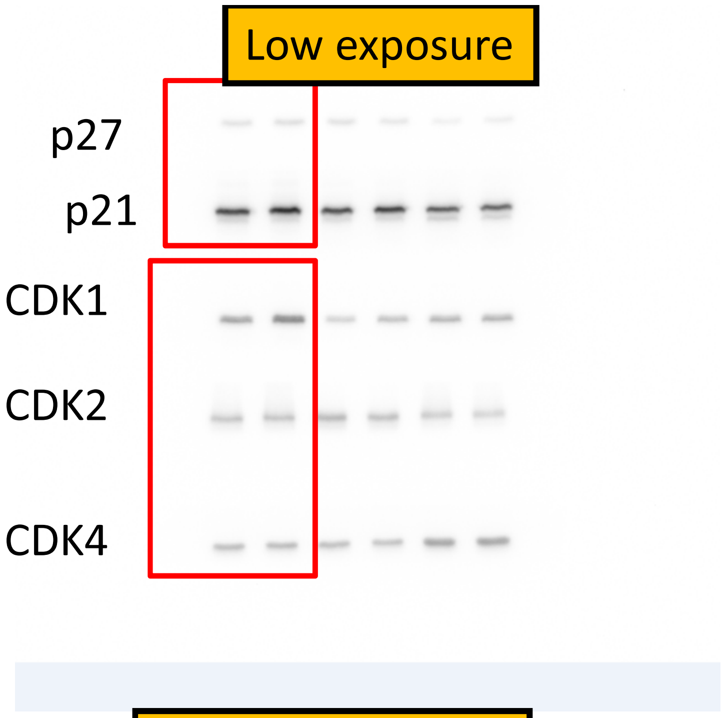

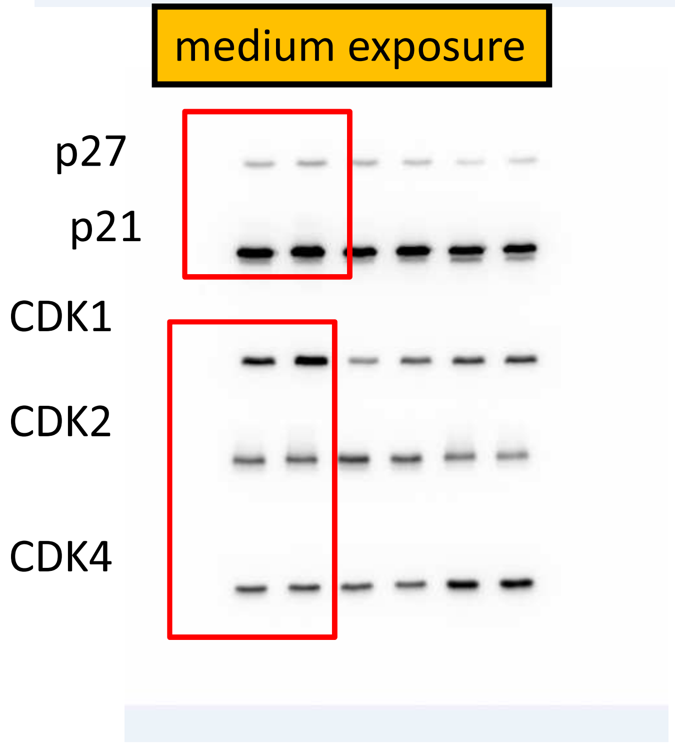

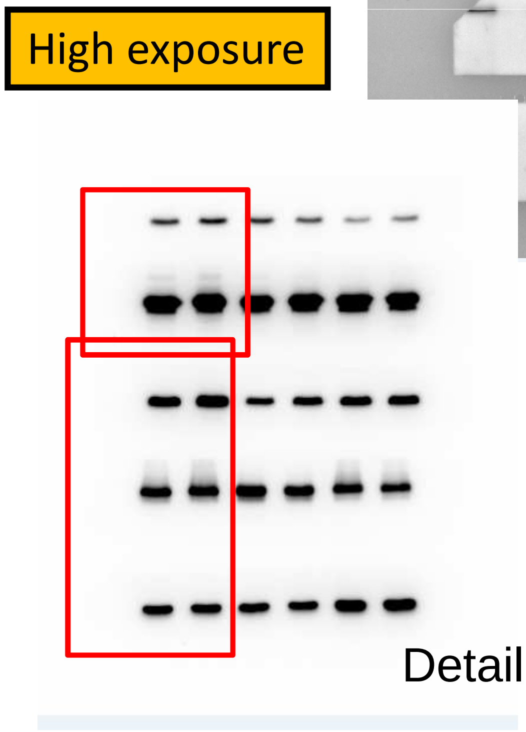

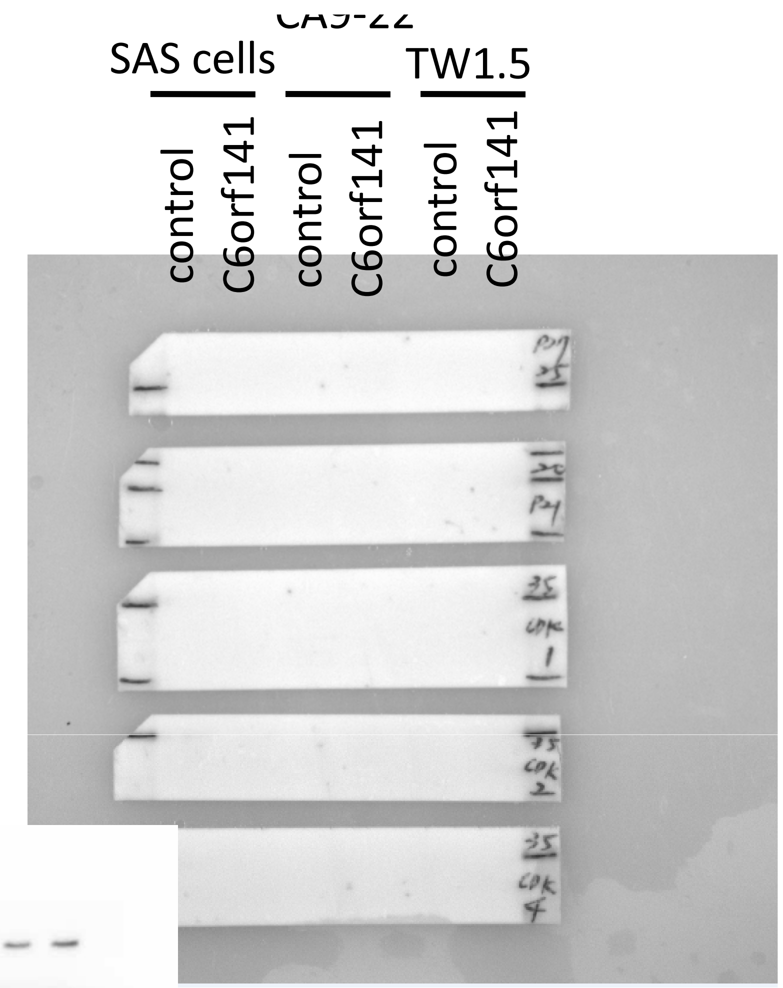

Detail information of figure 9c

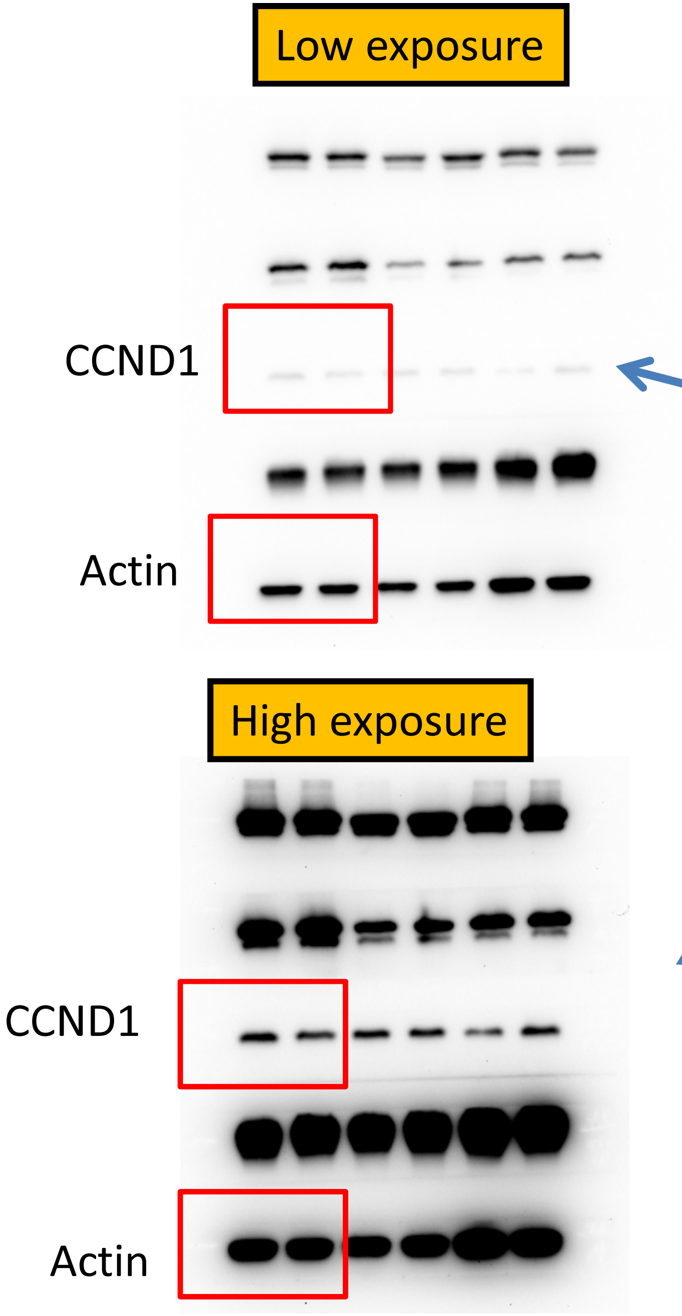

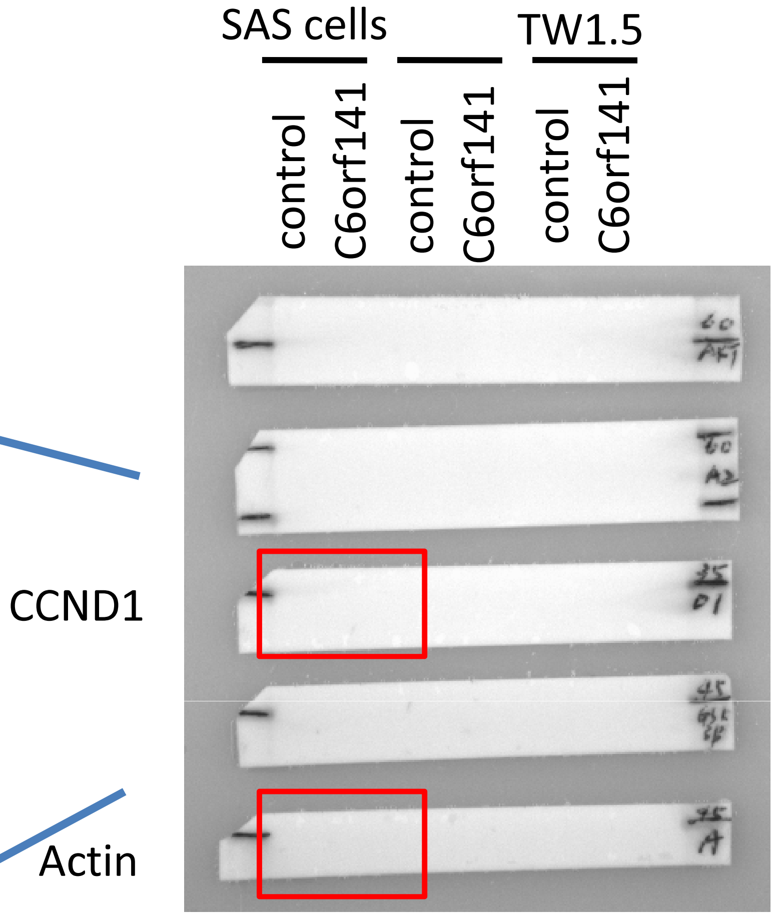

Detail information of figure 9c
